# Does Physical Activity Matter for the Mental Health of University Students during the COVID-19 Pandemic?

**DOI:** 10.3390/jcm9113494

**Published:** 2020-10-29

**Authors:** Aleksandra M. Rogowska, Iuliia Pavlova, Cezary Kuśnierz, Dominika Ochnik, Ivanna Bodnar, Petro Petrytsa

**Affiliations:** 1 Institute of Psychology, University of Opole, 45-052 Opole, Poland; 2 Theory and Methods of Physical Culture Department, Lviv State University of Physical Culture, 79007 Lviv, Ukraine; pavlova.j.o@gmail.com (I.P.); ivannabodnar@ukr.net (I.B.); 3 Faculty of Physical Education and Physiotherapy, Opole University of Technology, 45-378 Opole, Poland; c.kusnierz@po.edu.pl; 4 Faculty of Medicine, University of Technology, 40-555 Katowice, Poland; dominika.ochnik@gwsh.pl; 5 Department of Physical Education, Ternopil Volodymyr Hnatiuk National Pedagogical University, 46027 Ternopil, Ukraine; petrytsa@tnpu.edu.ua

**Keywords:** anxiety, depression, PHQ-9, GAD-7, physical activity (PA), undergraduates, university students

## Abstract

Research indicates that university and college students are at higher risk of experiencing mental health problems than other populations. This study aims to examine the relationship between Physical Activity (PA) and the mental health of Ukrainian university students during the Corona Virus Disease 2019 (COVID-19) pandemic lockdown. The conventional sample consisted of 1512 students from 11 Ukrainian universities, with a mean age of 20 years (M = 20.06, SD = 3.05) and 69% of whom were female. The cross-sectional online survey was disseminated through the most popular social media channels in Ukraine (i.e., Facebook, Viber, Telegram) and included the Generalized Anxiety Disorder (GAD-7) scale to measure anxiety and the Patient Health Questionnaire (PHQ-9) to assess depression. Data were collected from 14 May to 4 June 2020 during the COVID-19 pandemic outbreak in Ukraine. Among university students, 43% were engaged in PA ≥ 150 min weekly, 24% met the criteria of GAD, and 32% met the criteria of depression. More students were involved in PA before the COVID-19 outbreak than during the national lockdown. Students with anxiety and depression were almost two times less likely to engage in PA than their counterparts without mental health disorders. The inactive group had higher scores of anxiety and depression than the physically active group. The relationship of PA with anxiety and depression was statistically significant but weak during the COVID-19 pandemic.

## 1. Introduction

### 1.1. Coronavirus Disease in Ukraine

This study examines the mental health of Ukrainian university students during the first two months of the Corona Virus Disease 2019 (COVID-19) outbreak. On 3 March 2020, Ukraine announced the first confirmed case of the coronavirus disease. On 30 April 2020, approximately 10,860 cases of COVID-19 were recorded. Merely one month later (on 31 May 2020), the number of cases was confirmed as 24,012. The Cabinet of Ministers of Ukraine adopted a resolution on preventing the spread of the coronavirus COVID-19 in Ukraine on 11 March 2020. Following the resolution, quarantine was enforced in the territory of Ukraine from 12 March 2020. Students were prohibited from attending or visiting any type of educational establishment and mass events were canceled and banned. On the same day, the Ministry of Education and Science published a letter stating the need to comply with the rules of the quarantine. As a consequence, kindergartens, schools, colleges, and universities were closed, and e-learning using remote technologies was implemented. Lockdown was introduced in all regions of Ukraine. State borders were closed, and a minimum period was granted for the return of citizens who were abroad at that time. On 16 March, Ukraine closed international air, rail, and bus services. On 18 March, domestic rail, air, and bus services were suspended. In addition, intercity and interregional passenger traffic, pedestrian traffic, and the use of public transport were also significantly restricted. Social distancing and the wearing of face masks were introduced as obligatory requirements.

According to government decrees in early April, it was forbidden to visit parks, squares, recreation areas, coastal areas, sports areas, and children’s playgrounds. Moving in groups of more than two people was also banned. Citizens were not allowed to stay outside their homes or be on the streets without documents. The police and the military were involved in monitoring the self-isolation regime. The penalty for the violation of quarantine conditions was a fine or imprisonment. A five-step program for quarantine alleviation was implemented after 22 May 2020, dependent on the epidemiological situation in each region. In June 2020, educational institutions’ work was only carried out remotely. Social distancing and the wearing of facemasks were still required.

### 1.2. Mental Health during the COVID-19 Pandemic

Although a lockdown increases the chance of effectively dealing with COVID-19, it also contributes to a decrease in people’s well-being [1,2,3,4], invoking several adverse emotional reactions such as anger, fear, confusion, irritability, frustration, elevated stress, insomnia, and nervousness [1,4,5]. Thousands of people have lost their work and socio-economic status during the COVID-19 pandemic. Numerous studies have reported an increase in anxiety and depression among people around the world [5,6,7,8,9,10,11,12,13,14,15,16,17,18]. A review study indicated that, up to the time of its publishing, the prevalence of anxiety and depression ranged between 16–28% of the Chinese population during the COVID-19 pandemic [13]. Furthermore, it was found that women were at higher risk of coronavirus-related mental health disorders than men [16,19,20,21,22,23]. Research also indicated that younger adults were at higher risk of anxiety, depression, and alcohol use than adults with an average age and above [6,9,10,19,22,23].

A growing body of literature has recently focused on examining mental health among undergraduates [18,20,21,24,25,26,27,28,29]. Those studies suggest that university and college students experience higher rates of anxiety than it has been reported in other population groups [18,20,21,24,28]. However, a disparity in the prevalence of mental health disorders was also noted between particular studies, dependent on cross-cultural differences and the measurement methods used to assess anxiety and depression. When a seven-item General Anxiety Disorder (GAD-7) scale was used, Cao et al. [24] found that approximately 30% of Chinese students (*n* = 7143) reported symptoms of mild (21.3%), moderate (2.7%), and severe (0.9%) anxiety during the COVID-19 pandemic. In research by Feng et al. [25], anxiety was reported for 32% (in the GAD-7) of Chinese students, and depression was found in 28%, using a nine-item Patient Health Questionnaire (PHQ-9). The prevalence of general anxiety disorder (using GAD-7) among Polish university students (*n* = 914) was 67%, including 32% with mild, 21% with moderate, and 14% with severe symptoms. Among nursing students, the prevalence of moderate and severe anxiety (using the GAD-7) was 42.8% and 13.1%, respectively, during the third week of the national lockdown in Israel [28]. Sallam et al. [21] found a mean anxiety score of 8.4 (assessed using GAD-7), with statistically significant higher scores among males (M = 7.7) compared to females (M = 8.6).

It is important to note that the high risk of anxiety and depression disorders was noted among university and college students before the pandemic started, and this trend has been observed in research for years. A review of previous studies conducted before the COVID-19 pandemic showed that depression was present on average in 30.6% of undergraduates [30,31], with significantly higher rates among women. Studies revealed that anxiety has been diagnosed in 12–43% of college and university students [31,32,33]. Thus, there is a need to develop and integrate various prevention and intervention programs at university campuses to cope with stress, anxiety, and depression. One of the ways to reduce the levels of anxiety and depression is engagement in healthy behavior, such as physical activity (PA).

### 1.3. Physical Activity and Health

The beneficial effect of PA on mental health is well-documented in many studies e.g., [34,35,36,37,38,39,40]. Regular exercise improves sleep, mood, endurance, and cardiovascular fitness; reduces cholesterol, body weight, tiredness; and helps boost stress relief, energy, and interest in sex. Therefore, exercise is recommended as a prescription for patients with various conditions including respiratory system diseases [41]. Recent research found that enhanced aerobic capacity could improve immune functions, decrease COVID-19 severity, and may help prevent infection [42].

Although home-based physical training was recommended during the COVID-19 lockdown [43,44,45], some research suggested that the intensity of PA decreased among people in Asia, America, Africa, and Europe [23,34,46,47]. On the other hand, a general increase in PA was observed in Belgium [48] and Canada [49] during the coronavirus outbreak. This inconsistency regarding the changes in the intensity of PA during the pandemic should be monitored, particularly in the groups of the population at higher risk of mental health disorders such as university and college students.

Here, we examine the relationship between PA and both anxiety and depression in a large sample of university students in Ukraine during the national lockdown. Previous research conducted prior to the COVID-19 pandemic indicates that 58% of college students met the WHO physical activity guidelines (i.e., ≥150 min per week of PA) in Canada [50]. Furthermore, Ghrouz et al. [51] showed that Indian college students with moderate and high PA levels (49% of the total sample) reported significantly lower anxiety and depression scores than their counterparts with a low PA level. To the best of our knowledge, only one study has explored the PA level and mental health of college students during the COVID-19 pandemic so far [18]. That study indicated that approximately 56% of Chinese students were engaged in moderate and high levels of PA during the coronavirus outbreak [18]. It was also shown that PA alleviated negative emotions and depression.

The following research questions were posed in the present study: (1) What is the prevalence of anxiety and depression disorders during the coronavirus pandemic in a sample of university students in Ukraine? (2) How many Ukrainian undergraduates meet the PA level consistent with the WHO recommendation (≥150 min per week) during the COVID-19 lockdown? (3) Has the level of PA changed from before the pandemic? (4) What is the association between PA, anxiety, and depression in the study sample? Due to differences between the sexes found in previous studies on PA, anxiety, and depression during the COVID-19 pandemic, sex will be controlled in this study as a covariate.

We formulated the following hypotheses:

1. Based on previous research, we expected approximately one-third of university students to have suffered from various forms of depression and anxiety, experiencing moderate to severe symptoms [6,9,10,13,16,17,18,20,21,25,52].
2. We expected approximately half of the university students to meet the recommended criteria of sufficient PA level (≥150 min per week) during the COVID-19 pandemic, as previously suggested [18].
3. We expected that the level of PA would have changed during the COVID-19 pandemic compared to the typical PA level before the coronavirus outbreak [23,34,46,47,48,49].
4. We expected physically active university students to demonstrate lower scores in anxiety and depression than their inactive counterparts [18,51]. The appropriate level of PA (≥150 min/week) can predict levels of anxiety and depression.

## 2. Materials and Methods

### 2.1. Participants

The sample consisted of 1512 students whose age ranged between 18–51 (M = 20.06, SD = 3.05), most of whom were female (*n* = 1038, 68.65%). The target population was comprised of undergraduates from 18 Ukrainian universities located in 11 cities: Kyiv (National Aviation University, National University of Food Technologies), Kharkiv (Yaroslav Mudryi National University, Kharkiv National Kotlyarevsky University of Arts), Lviv (Lviv Polytechnic National University, Ivan Franko National University of Lviv, Lviv State University of Physical Culture named after Ivan Boberskyj), Drohobych (Drohobych Ivan Franko State Pedagogical University), Ternopil (Ternopil Volodymyr Hnatiuk National Pedagogical University), Lutsk (Lesya Ukrainka Eastern European National University, Lutsk National Technical University), Mykolaiv (Petro Mohyla Black Sea National University, Mykolaiv V.O.Sukhomlynskyi National University), Poltava (National University “Yuri Kondratyuk Poltava Polytechnic”), Cherkasy (Bohdan Khmelnytsky Cherkasy National University), Chernivtsi (Yuriy Fedkovych Chernivtsi National University), and Ivano-Frankivsk (Vasyl Stefanyk Precarpathian National University). Territorially, the study covered 10 of the 27 Ukrainian regions. Students represented various university majors at various levels of higher education. Table 1 outlines the demographic characteristics of the sample (age, gender, faculty, and level, year, and type of study).

The total number of students in Ukraine is about 1.5 million people. According to state statistics in Ukraine [53], the largest group consists of state institutions of higher education (universities, academies, institutes, colleges, technical schools, colleges), amounting to 456 institutions (381 when excluding separate units); there are 191 private institutions 191 (140 when excluding separate units). The number of institutions of the highest level of accreditation (universities, institutes, academies) was 282, with 78 private institutions at this level. Of the total number of students, 400,000 are in extramural study and 106,000 are in private higher education institutions. Approximately 90% of students in universities, institutes, and academies are aged 17–24 years.

There are more females in higher education. This trend at universities has been stable over the past ten years [53]. With regard to study majors, constant changes began in higher education from 2015. The available statistics [53,54] offer only a partial understanding of the structure of student distribution by field of study. The majority of students choose social sciences (38.3%); natural sciences (6.4%); engineering, architecture, and construction (19–21%); education and pedagogy (8%); service industries (6.9%); and health and social security (8%).

In the present survey, we tried to avoid questions concerning ethnicity (Ukrainian, Russian) due to the military actions in eastern Ukraine provoked and initiated by the Russian Federation. The questionnaire was written in Ukrainian and was intended only for local students who spoke Ukrainian at the native speakers’ level. According to national statistics [55], 96.2% of young people aged 18–29 consider themselves ethnic Ukrainians, 2.8% Russians, and 0.7% of other nationalities. For 2019, the number of international students did not exceed 5%. Among students of other nationalities (foreign students) studying in Ukraine are citizens from India (*n* = 14,958), Morocco (*n* = 7390), Azerbaijan (*n* = 6228), Turkmenistan (*n* = 5033), Nigeria (*n* = 3552), Egypt (*n* = 3412), Turkey (*n* = 3254), China (*n* = 2721), Israel (*n* = 2460), and Georgia (*n* = 2397) [53]. Most international students traditionally choose Ukrainian medical colleges and universities, but we did not collect data from students in this type of higher education.

### 2.2. Measures

#### 2.2.1. Exposure to COVID-19

Exposure to COVID-19 was assessed based on eight questions about the consequences of the coronavirus: (1) Have you experienced symptoms that could indicate coronavirus infection? (2) Have you been tested for coronavirus? (3) Were you hospitalized by coronavirus? (4) Did you have to be in strict quarantine for at least 14 days, that is, in isolation from loved ones because of the coronavirus infection? (5) Has anyone in your family or friend group been infected with coronavirus? (6) Have any of your relatives died of coronavirus? (7) Have you or a loved one lost their job because of coronavirus? (8) Are you currently experiencing a worsening of your functioning or economic status due to the effects of the coronavirus pandemic? Individuals answered each of these questions with 1 = Yes and 0 = No.

#### 2.2.2. Perceived Impact of Coronavirus

The perceived impact of COVID-19 on the student’s life was measured using five statements. Participants used a 5-item Likert scale (from 1 = Strongly disagree, to 5 = Definitely agree) to express how much they were afraid that the current situation associated with the coronavirus pandemic (COVID-19) may negatively affect their life in each of the following five areas: (1) Completing the semester and graduation; (2) finding a job and professional development; (3) financial situation (e.g., subsistence during studies); (4) relationships with loved ones and family; (5) relations with colleagues and friends. Next, the scores obtained from the five items were summarized to give a total score of perceived coronavirus impact (PCI). Higher scores indicated greater coronavirus-related concerns. The internal reliability of the scale was acceptable with Cronbach’s α = 0.65.

#### 2.2.3. Physical Activity

Physical activity during the coronavirus-related lockdown was assessed using the following question: “How many days a week did you exercise physically or pursue sports activities at home or away from home, at the university, in clubs or at the gym, in the last month?”. Participants answered this question on an eight-point scale (from 0 = Not one day to 7 = Seven days a week). Next, the students answered the open question “how many minutes a day (on average) did you practice?”, writing an average number of minutes of PA per day. The next question was as follows: “How many days a week did you do physical exercise or sports activities at home or away from home, at the university, in clubs or at the gym, within a month before the general coronavirus quarantine?”, with the same scales used as in the answers for the previous questions. The number of days was multiplied by the number of minutes per day to calculate the PA level the previous week. Those students who performed 150 min weekly or more were included in the Active (A) group, whereas the Inactive (I) sample comprised those individuals who devoted less than 150 min per week to PA, according to the WHO recommendation [45].

#### 2.2.4. Anxiety

A seven-item generalized anxiety disorder (GAD-7) is a self-reported measure designed to screen for symptoms following the Diagnostic and Statistical Manual of Mental Disorders (DSM-IV) criteria [56]. People rated, on a 4-point Likert scale (0 = Not at all, 1 = Several days, 2 = More than half the days, and 3 = Nearly every day), how often they experienced anxiety symptoms in the past 2 weeks. The GAD-7 scores ranged between 0–4, indicating no or minimal anxiety, between 5–9 mild, between 10–14 moderate, and between 15–21 severe GAD [57]. Scores above 10 points indicated an anxiety disorder. In this study, the Cronbach’s α for the GAD-7 was 0.90.

#### 2.2.5. Depression

A patient health questionnaire (PHQ-9) was used to measure symptoms of depression among university students. PHQ-9 consisted of nine items, corresponding to the DSM-IV diagnostic criteria [58,59]. Participants answered questions about how often over the past two weeks they had been bothered by nine depression symptoms, using a Likert-like response scale ranging from 0 = Not at all, to 3 = Nearly every day. The PHQ-9 scores may be interpreted as normal between 0–4, mild major depressive disorder between 5–9, moderate between 10–14, moderately severe between 15–19, and severe between 20–27 [58]. At a score of 10 or above, it is recommended to screen for a major depressive disorder [58,60,61]. In this study, the Cronbach’s α for the PHQ-9 was 0.85.

#### 2.2.6. Demographics

The survey also included eight questions related to demographic variables. The questions referred to age, gender, place of residence (village, small town, big city, and urban agglomeration), field of study, study major, level of study (i.e., first degree, secondary studies, uniform five-year studies, doctoral studies), year of the study (ranging 1–5), and mode (i.e., stationary or extramural). Table 2 presents the frequency of the given response category in the study sample.

### 2.3. Procedure

A cross-sectional study was performed using a self-reported Google Forms survey. This study was part of an international research project preregistered in the Open Science Framework (OSF) [62]. Researchers collaborated with students’ trade unions and student government organizations to disseminate an invitation to participate in the study among the students of target universities. The invitation was distributed by Facebook groups, Viber groups, and Telegram channels. The data were collected from 14 May to 4 June 2020 and 99% of the responses were dated between 14 May and 1 June. The first website of the Google Forms survey consisted of information about the study and an informed consent form. All students were informed about the purpose of the study and participation was voluntary. They were also assured about the anonymity and confidentiality of the survey and that they may refuse to participate in the study at any time. Initially, 1542 students visited the website with the survey. However, 30 undergraduates did not agree to participate in the study. Ultimately, 1512 respondents gave their informed consent and completed the questionnaire (98.1% response rate). This study was approved by the Institutional Research Board (IRB) of the Lviv State University of Physical Culture (No. 4/2020.04.01) and was conducted according to the principles of the Declaration of Helsinki.

#### Statistical Analysis

Descriptive statistics, such as the mean (*M*), standard deviation (SD), range, standard error (SE), and 95% confidential intervals (CI) with lower limit (LL) and upper limit (UL) calculated for the total sample. Pearson’s χ^2^ test of independence was performed to find an association between physical activity (PA) before and during the coronavirus pandemic. A two-way ANOVA was performed to examine sex and PA differences (considered as dichotomous discrete variables) in anxiety (continuous variable). The effect size was measured by partial eta-squared (η*_p_*^2^), which describes the ratio of variance explained in the dependent variable by a predictor in the ANOVA model. The Tukey’s honest significant difference test (HSD) was performed to examine post hoc differences between mean scores. The associations between mental health and PA were examined using binominal logistic regression analysis. The relationships between anxiety, COVID-19 impact, physical activity, and sex were examined using Pearson’s correlation analysis. Finally, multiple linear regression analysis was conducted to find the moderation effect of physical activity and sex on the relationship between COVID-19 impact and anxiety. All analyses were performed using Statistica 13.3. (Statsoft Polska Sp. z o.o., Kraków, Poland)

## 3. Results

### 3.1. Prevalence of Anxiety and Depression among Undergraduates during the COVID-19 Pandemic

Anxiety criteria (GAD-7 ≥ 10) were met by 360 undergraduates (24%), while depression (PHQ-9 ≥ 10) was found in 479 university students (32%). Overall, people with clinically significant depression and/or anxiety totaled 554 (37%), whereas 958 (63%) of people did not display significant symptoms of mental disorder. In the study sample, individuals meeting exclusively anxiety criteria numbered 75 (5%), those exclusively with depression criteria numbered 194 (13%), and those with dual depression and anxiety symptoms totaled 285 undergraduates (19%). The contingency table showed that the difference between people with singular and dual symptoms was statistically significant, χ^2^ (1) = 492.28, *p* < 0.001, ϕ = 0.57. Descriptive statistics for anxiety and the impact of COVID-19 on students’ well-being are shown in Table 3.

### 3.2. Prevalence of PA before and during the COVID-19 Pandemic

Table 1 and Table 2 demonstrate the number and proportion of individuals exposed to the coronavirus disease and engaged in PA at a sufficient level (≥150 min/week, according to the WHO recommendations) before and during the national coronavirus quarantine. As shown in Table 2, most students reported being inactive both before and during the COVID-19 quarantine. During the COVID-19 outbreak, 43% of university students were engaged in PA at the desired level. In comparison, 46% of students reported being active before lockdown. A Pearson’s χ^2^ test of independence indicated that the differences between active and inactive participants significantly changed over time; χ^2^ (1) = 254.93, *p* < 0.001, ϕ = 0.41. The same status of PA before and during the coronavirus pandemic was reported by 457 active individuals (30.22%) and 613 (40.54%) inactive individuals. People active before the quarantine but becoming inactive during the COVID-19 pandemic numbered 246 (16.27%). Conversely, those inactive before the pandemic outbreak but becoming active during the coronavirus lockdown totaled 196 (12.96%). It suggests that students with a reduced level of PA during the COVID-19 pandemic outnumbered those becoming more active.

### 3.3. Differences in Anxiety and Depression between Physically Active and Inactive University Students

Results of the binominal logistic regression suggest that, during the COVID-19 pandemic, university students that met the clinical criteria for depression (the PHQ-9 scores ≥ 10) were 1.6 times less likely to engage in PA (≥150 min PA weekly) than those without clinically significant depression χ^2^ (1) = 19.04, OR = 1.64, 95% CI (1.31, 2.05), B = 0.49, SE B = 0.11, t(1510) = 4.32, *p* < 0.001, Wald χ^2^ = 18.67. Undergraduates that met the clinical criteria for anxiety (the GAD-7 scores ≥ 10) were 1.7 times less likely to exercise than their counterparts without clinically significant anxiety disorders, χ^2^(1) = 17.98, OR = 1.69, 95% CI (1.32, 2.17), B = 0.53, SE B = 0.13, t(1510) = 4.18, *p* < 0.001, Wald χ^2^ = 17.47. Undergraduates with the dual clinical criteria of depression and anxiety (scores in the PHQ-9 ≥ 10 and GAD-7 ≥ 10) were 1.9 times less likely to be physically active than people without clinically significant mental health symptoms, χ^2^ (1) = 22.84, OR = 1.95, 95% CI (1.47, 2.58), B = 0.67, SE B = 0.14, t(1241) = 4.68, *p* < 0.001, Wald χ^2^ = 21.89.

**Figure 1:**
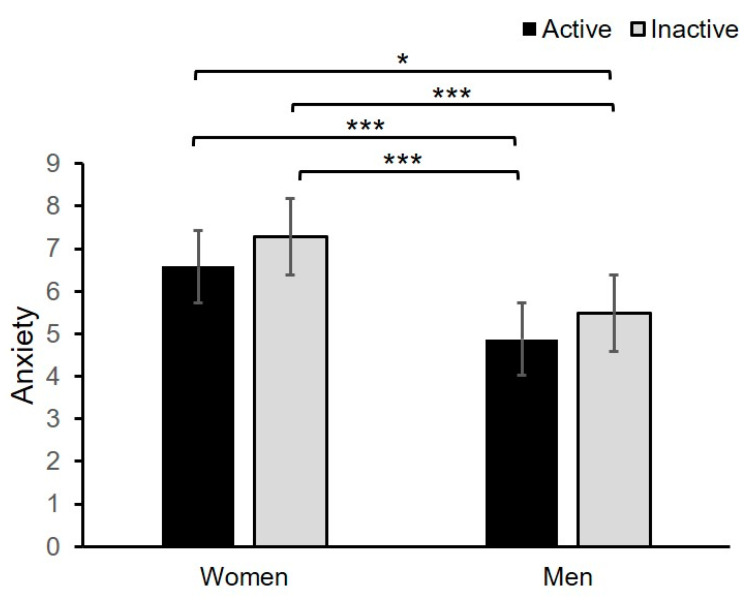
Mean anxiety scores for groups of undergraduates differing in sex (women, men) and engagement in physical activity (active, inactive). Error bars show standard errors. * *p* < 0.05, *** *p* < 0.001.

Table 4 and Figure 1 demonstrate some results of the two-way ANOVA test with anxiety as the dependent variable and sex (female, male) and PA groups (inactive, active) as the independent variables. A significant effect was found for sex and PA separately (Table 4). The Tukey HSD post hoc test showed that females scored significantly higher in anxiety than males and physically inactive female students (those who were engaged in PA less than 150 min weekly during the coronavirus pandemic). Levels of anxiety were similar in physically active and inactive female students. Physically active and inactive male university students did not differ in their anxiety levels. The effect of interaction between gender and PA was also not statistically significant.

**Figure 2:**
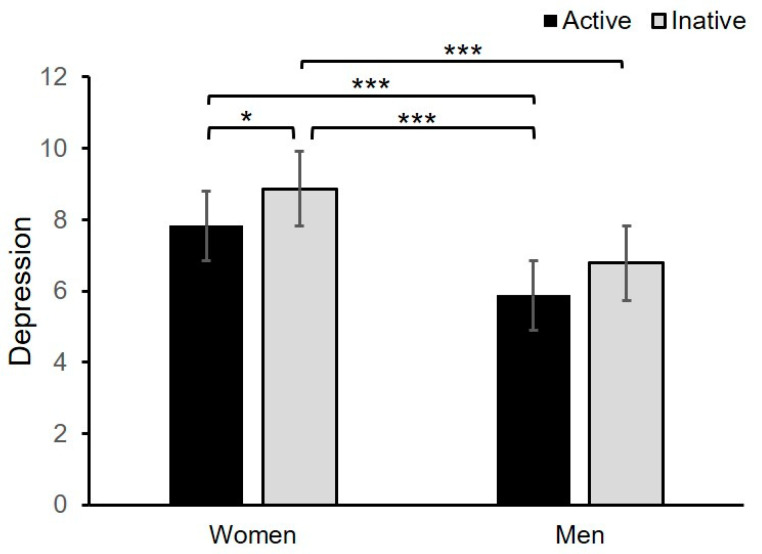
Mean depression scores for groups of undergraduates differing in sex (women, men) and engagement in physical activity (active, inactive). Error bars show standard errors. * *p* < 0.05, *** *p* < 0.001.

A two-way ANOVA was conducted for depression as a dependent variable and sex (female, male) and PA groups (inactive, active) as independent variables (see Table 5 and Figure 2 for more details). Both sex and PA were statistically significant factors for depression but without an interaction effect. The Tukey HSD post hoc test showed that physically active women differ significantly in depression to inactive female students, as well as active and inactive males. Significant differences were also found between inactive women and active men.

### 3.4. Predictors of Anxiety and Depression

Correlation analysis showed that anxiety was positively associated with exposure to COVID-19 (r = 0.25, *p* < 0.001) and the perceived coronavirus impact (PCI) on students’ well-being (r = 0.16, *p* < 0.001). Hierarchical multiple linear regression was conducted separately for anxiety and depression as an explanatory variable and sex, exposure to COVID-19, the impact of COVID-19 on well-being, and physical activity (PA) as predictor variables. Both regression models were tested in a preliminary analysis to ensure that they met the assumptions of residual normality, linearity, homoscedasticity, and non-collinearity (using VIF < 10). All criteria were met. As presented in Table 6, all variables were found to be significant predictors of anxiety. Sex alone explained about 3% of anxiety variability, F(1, 1510) = 48.41, *p* < 0.001. The negative correlation found between sex and anxiety indicated that females (coded as “0”) presented higher anxiety levels than males (coded as “1”). When exposure to COVID-19 was included in the regression in the second step, the model explained 9% of anxiety variability, F(2, 1509) = 73.23, *p* < 0.001. The change in variance explained was 6%, F(1, 1509) = 95.04, *p* < 0.001. The third model of regression included the impact of COVID-19 on students’ well-being. The variability explained significantly increased to 13% (F(3, 1508) = 74.50, *p* < 0.001), with 4% of change in variance explained (F(1, 1508) = 70.30, *p* < 0.001). The fourth step of regression analysis included PA, which significantly changed the variance explained to 14% (F(4, 1507) = 61.92, *p* < 0.001). The variability explained changed significantly by about 1% (F(1, 1507) = 21.21, *p* < 0.001). The negative association between PA and anxiety indicates that those university students who spent less than 150 min doing PA every week during the COVID-19 pandemic experienced higher levels of anxiety than their counterparts who engaged in more than 150 min PA per week.

Depression was found to be positively associated with exposure to COVID-19 (r = 0.23, *p* < 0.001) and PCI (r = 0.23, *p* < 0.001). Hierarchical multiple regression analysis showed that all variables included in the model were significant predictors of depression (Table 7). Similar to the previous analysis, sex explained about 3% of depression variability (F(1, 1510) = 54.01, *p* < 0.001), with higher depression among female students than male. In the second step, gender and exposure to COVID-19 explained for 8% of anxiety variability, F(2, 1509) = 70.46, *p* < 0.001. The change in variance explained equaled 5%, F(1, 1509) = 83.95, *p* < 0.001. When PCI was included in the third regression model, the percentage of variability explained significantly increased to 12% (F(3, 1508) = 71.29, *p* < 0.001), with 4% of change in variance explained (F(1, 1508) = 66.81, *p* < 0.001). The fourth model of regression included PA, which significantly changed the variance explained to 15% (F(4, 1507) = 66.64, *p* < 0.001). The variability explained changed significantly by about 3% (F(1, 1507) = 46.26, *p* < 0.001). In comparison with the hierarchical regression model for anxiety, PA seems more important for depression than for anxiety.

## 4. Discussion

### 4.1. Mental Health in Ukrainian University Students

This study aimed to examine PA’s relationship with the mental health of university students during the COVID-19 pandemic. Results suggest that 37% of the study sample suffered from moderate to severe anxiety and/or depression. Symptoms of depression were significantly more frequently found than symptoms of anxiety. In addition, more people had dual clinically significant mental disorders (anxiety and depression simultaneously) than those who met the singular criteria of the PHQ-9 and GAD-7. Approximately one-quarter of students may suffer from general anxiety disorders, while nearly one-third may be affected by depression. Furthermore, almost one-fifth of university students manifested symptoms of anxiety and depression concurrently. This means that the vast majority of individuals with anxiety symptoms experienced the comorbidity of depression.

The hypothesis that approximately one-third of the university student population suffered from various forms of depression and anxiety (from moderate to severe symptoms) was confirmed in this research regarding depression, but anxiety levels were lower than expected. The present result is consistent with the study of Feng et al. [25] but differs from certain previous studies performed in the student population during the COVID-19 pandemic [18,20,21,28]. The prevalence of depression in the present study sample was 32%. In comparison, between 23% [18] and 32% [25] of depression was reported among college and university students in China. General anxiety disorder was found in approximately 24% of Ukrainian university students, as compared to 4% [24], 28% [25] and 45% [18] among Chinese college students, 35% of Polish university students [20], and 56% in nursing students from Israel [28]. The mean anxiety score was 6.43 in the present sample of Ukrainian university students, while Jordanian students reported a mean anxiety score of 8.4 [21]. Anxiety in the present study was lower than in most previous studies reported for both students and general populations.

A review indicated that the prevalence of anxiety and depression ranged between 16–28% of the Chinese population during the COVID-19 pandemic at the time of publishing [13]. However, differences between particular studies can be seen in mental health symptoms during the COVID-19 pandemic. More specifically, moderate to severe anxiety was noted in 29%, whereas moderate to severe depression was reported in 16% of the population living in China during the COVID-19 pandemic outbreak [16,17]. Among Chinese people younger than 35, 38% reported general anxiety disorder (GAD), and 22% reported depression symptoms [9,10]. Ahmed et al. [6] found 19% of people suffered from anxiety and 27% suffered from symptoms of depression ranging from moderate to severe among 1074 Chinese people living in Hubei province. The prevalence of depression, anxiety, and comorbidity of depression and anxiety was 48.3%, 22.6%, and 19.4%, respectively, among people from Wuhan province in China [52]. Approximately 23% of individuals from Cyprus reported moderate to severe anxiety symptoms, and 9% reported moderate to severe depression symptoms [22].

The differences between particular studies may be determined, besides cross-cultural differences, by the various methods used to measure anxiety and depression, the distinct quality of the study, and the various cut-off criteria for diagnosing anxiety and depression. A recent meta-analysis indicated that the prevalence of depression assessed using the PHQ-9 scores ≥10 are often overestimated when compared to a diagnosis of major depression on the base of Structured Clinical Interview for Diagnostic and Statistical Manual of Mental Disorders (SCID) [63]. On the other hand, the PHQ is a self-reported screening questionnaire used to detect a risk of depression by an average non-clinician person, so overestimating seems to be a desired feature in this case. Another systematic review and meta-analysis [64] showed that PHQ-9 has acceptable diagnostic properties for major depressive disorders (with cut-off points between 8–11), and diagnostic accuracy was reasonably consistent despite the clinical heterogeneity of the included studies. Most likely, certain anxiety and depression measures present better sensitivity, specificity, and reliability than others. A previous meta-analysis showed a wide range of differences in the global prevalence of anxiety among medical students, which may also be related to cross-cultural differences [65]. Moreover, research was conducted at different stages of the COVID-19 outbreak. Many countries in the world introduced several coronavirus-related restrictions in traveling; shopping; access to education, medical and social services; outdoor physical activity; gatherings; and meetings with friends and family. However, these restrictions were changed systematically within each country. As a consequence, a reliable comparison of mental health during the COVID-19 pandemic may be very difficult to achieve.

Consistent with most of the previous research, the analysis of variance showed that female Ukrainian university students score higher than men in anxiety and depression [16,18,19,20,21,22,23]. However, it is essential to note that these differences are rather weak, since the effect size was very small (η*_p_*^2^ = 0.03), and sex was not found to be a predictor for both anxiety and depression. Previous research indicated that the fear of the COVID-19 disease was positively related to a younger age, being female, and those more likely to keep smoking and drinking alcohol among medical students in Vietnam [26]. Among Jordanian university students, higher anxiety was reported in females, individuals with the lowest monthly income, those with less coronavirus knowledge, and those who believed the disease was part of a conspiracy [21].

The present results do not differ substantially from previous research conducted before the coronavirus pandemic outbreak [30,31,32,33]. It is confirmed that university and college students experience consistently higher rates of mental health disorders than other populations, which may be slightly elevated by the COVID-19 pandemic and lockdown restrictions. The findings from the regression analysis suggest that exposure to COVID-19 and individual differences in the perception of the coronavirus impact (PCI) showed significant but weak associations with anxiety and depression, so other factors may be more important in explaining mental health among Ukrainian university students.

However, the COVID-19 pandemic has given rise to many new concerns among the academic population. Besides the usual restrictions, university students have to contend with virtual learning during the COVID-19 pandemic. Online learning was not available to many students due to the lack of computers or internet access. During the pandemic, neither the students nor the teachers had been trained in the use of large-scale web-based teaching platforms and technology. Teachers were not familiar with the new online learning methodology. They had to change learning plans to adapt to the unique situation rapidly. Thus, the virtual classes were stressful for both teachers and students [18,66,67,68,69].

Furthermore, most of the students lost their part-time jobs which supported them while studying and offered them financial independence [70]. Adults who lived in dormitories had to return to their family homes and be dependent on their parents. During the first months of the outbreak of the pandemic, a prevalence of uncertainty regarding the near future, examinations, completing classes and finishing studies, the financial situation, and housing and social situation was evident [69]. Cao et al. [24] showed that economic factors affected daily life during the COVID-19 pandemic and that delays in academic activities were positively related to anxiety symptoms among Chinese college students. Furthermore, Qiu et al. [19] suggested that young adults tend to obtain a large amount of information from social media, which may elevate stress. Overall, the SARS-CoV-2 was perceived as a moderately dangerous disease by most students [21].

### 4.2. Relationship between PA and Mental Health

The present study suggests that 43% of university students were physically active during the coronavirus lockdown, according to the WHO recommendation (≥150 min/week of PA) [45]. A previous study found that 56% of Chinese college students were physically active at moderate or vigorous levels during the national quarantine [18]. The results suggest that the sample of Ukrainian students may be less involved in PA than the group of Chinese undergraduates. However, the previous research [18] used different measurement methods (i.e., a seven-item International Physical Activity Questionnaire—IPAQ) to assess weekly PA during the last two weeks according to three categories: light, moderate, and vigorous. Thus, the previous and present studies are not fully comparable.

Furthermore, the number of active students decreased significantly in comparison to the situation before the COVID-19 outbreak in Ukraine, which is consistent with some previous studies [23,34,46,47]. Stanton et al. [23] showed that negative physical activity changes are associated with increased depression, anxiety, and stress symptoms. Research indicates that exercise withdrawal may consistently result in an increase in depressive symptoms and anxiety [71].

Consistent with other research [18,51], this study indicates that there is a significant and inverse relationship between PA and anxiety and depression during the COVID-19 pandemic. A longitudinal survey showed that PA directly alleviated general negative emotions in college students during the peak time of the COVID-19 outbreak in China [18]. The volume of moderate-to-vigorous leisure-time PA (MVPA) was also positively associated with mental health and negatively related to the symptoms of anxiety and depression among post-secondary students aged between 16–24 years [72]. Furthermore, increasing PA during the COVID-19 lockdown was related to lower anxiety and improved well-being among individuals who were inactive before the pandemic [49].

Ukrainian undergraduates with anxiety and depression symptoms are between 1.6 and 1.9 times less likely to be physically active than their counterparts without mental health problems. This result is consistent with the previous population study of DeMello et al. [73], which showed that individuals who do not engage in PA are two times more likely to exhibit symptoms of depression and anxiety compared with those who regularly pursue PA. Furthermore, the highest association with PA was found in this study for participants with a dual anxiety and depression diagnosis. Forte et al. [74] also found that severe depression was most common among adolescents with comorbid anxiety and low PA levels.

The anti-depressive and anxiolytic effects of physical activity on clinical and non-clinical populations were evidenced in a great number of studies e.g., [70,71,75,76]. A systematic review and meta-analysis showed that PA reduces depression by a medium effect and anxiety by a small effect, in non-clinical populations [77,78]. Previous meta-analyses found a moderate-to-strong negative relationship between PA and depression and inconsistent association of PA with anxiety (ranged between not statistically significant effect to a moderate beneficial effect of PA on anxiety) in clinical populations. Previous findings suggest that high PA significantly reduced the prevalence of depressive problems, but not anxiety disorders, among Chinese first-year college students [25]. In contrast, this study found a statistically significant but rather weak association of PA with anxiety and depression. The hierarchical regression model with PA, sex, exposure to COVID-19, and PCI as predictors explained only 14% of anxiety and 15% of depression variability. PA can solely explain 1% of anxiety variability and 3% of depression variability, as shown in the hierarchical regression model in the fourth step of the analysis. Thus, more research is necessary to explain the specific relationship between these variables.

### 4.3. Limitations of the Study

There are certain limitations to this study. First, the online recruitment method has several limitations. As the research was performed during the lockdown related to the COVID-19 pandemic, all students used remote web-based technologies to learn in virtual educational platforms. An online survey was the only way to perform research on student well-being during the general quarantine. However, the invitation was distributed via Facebook groups, Viber groups, and Telegram channels, so students who do not use these websites could not respond. The online survey results may not be consistent with “paper and pencil” questionnaires. Although the study sample was substantial, there were more female respondents. This proportion is consistent with sex prevalence in typical universities (although technical universities comprise more males). Further research may include a more balanced proportion of the sexes in the study sample. As this research was performed in universities exclusively, the results of this study may not be generalized into more technically focused types of universities. Moreover, this study’s results cannot be generalized into other adult populations, since participants in this study were exclusively university students. Some demographic variables, such as ethnicity, income, employment, marital status, or household members, were not included in the questionnaire. Thus, we do not know if these variables would be more useful as covariates in analyzing the relationship between PA and mental disorder. Further research should include more demographic variables related to the mental health of university students. We used a simple, dichotomous division of people into active and inactive groups according to the WHO recommendation of PA level over the past week. However, other survey questions concerning more details of the level of PA (mild, moderate, vigorous) or time of each of the types could be used in future research. Finally, due to the cross-sectional character of research, the results of the regression analysis should be treated with caution.

## 5. Conclusions

This study confirmed that a significant proportion of Ukrainian students experience high levels of anxiety and depression. Both anxiety and depression are, to some extent, related to gender and PA, but the relationship is rather weak though statistically significant. PA seems a relatively inexpensive and effective way to cope with the effects of the COVID-19 pandemic. Thus, the present research results may be used in the prevention and treatment of coronavirus-related mental health burdens on university campuses. Mailey et al. [79] recommended an internet-delivered physical activity intervention, which should be implemented with psychological counseling for college students suffering from anxiety and depression. Clemente-Suárez [80] suggests that the combination of psychological therapy with aerobic physical activity and nutritional recommendations can decrease anxiety and depression symptoms in just six sessions. WHO recommends a minimum of 150 min of physical activity weekly [45]. According to a recent study [18], just 108 min of mild PA, 80 min of moderate PA, or 45 min of vigorous PA per day is enough to prevent mental health disorders and maintain well-being during the COVID-19 pandemic, and this is a recommended intervention in university student populations. We propose the introduction of online PA training on e-learning platforms (e.g., Moodle, MS Teams, Zoom), conducted by a professional sports coach in the field of aerobic and anaerobic exercises varying in intensity level (light, moderate, and vigorous), to be chosen by students depending on their movement abilities and interests. Exercises should last a minimum of one hour and should be proposed online twice a day (morning and evening) each day. We believe that this PA training can be useful for all students in preventing mental health disorders. For university students with severe symptoms of anxiety and depression, supportive interventions can include physical exercises performed in conjunction with individual psychological online therapy.

## Figures and Tables

**Table 1:** Demographic characteristics of the study sample.

Demographic Variables	*n*	%
**Sex**		
Female	1038	68.65
Male	474	31.35
**Place of Residence**		
Village	494	32.67
Small town	535	35.38
Big city	434	28.70
Urban agglomeration	49	3.24
**Level of Study**		
Bachelor	1374	90.87
Master	127	8.40
Doctoral	11	0.73
**Branches of Study Science**		
Social Science	531	35.12
Formal Science	109	7.21
Natural Science	141	9.32
Health Science	731	48.35
**Year of Study**		
First	610	40.34
Second	346	22.88
Third	243	16.07
Fourth	222	14.68
Fifth	91	6.02
**Type of Study**		
Stationary	1417	93.72
Extramural	95	6.28

**Table 2:** Coronavirus-related variables.

Variable	*n*	%
**Physical Activity ≥** **150 min Weekly**		
Before Coronavirus Pandemic	703	46.49
During Coronavirus Pandemic	653	43.19
**Exposure To COVID-19**		
Symptoms of Coronavirus Infection	111	7.34
Testing for Coronavirus	34	2.25
Hospitalization Because of Coronavirus	8	0.53
Strict Quarantine For At Least 14 Days	122	8.07
Coronavirus Infection Among Close Relatives	83	5.49
Death of Coronavirus Among Close Relatives	7	0.46
Unemployment Because Of The Coronavirus	602	39.81
Worse Functioning or Lower Economic Status	1063	70.30
**Anxiety (GAD-7)**		
Normal (0–4)	618	40.87
Mild (5–9)	534	35.32
Moderate (10–14)	243	16.07
Severe (15–21)	117	7.74
**Depression (PHQ-9)**		
Normal (0–4)	476	31.48
Mild (5–9)	557	36.84
Moderate (10–14)	290	19.18
Moderately severe (15–19)	127	8.40
Severe (20–27)	62	4.10
**Neither Depression nor Anxiety Diagnosis (Scores ≤ 10)**	958	63.36
**Anxiety Only Diagnosis (GAD-7 ≥ 10)**	75	4.96
**Depression Only Diagnosis (PHQ-9 ≥ 10)**	194	12.83
**Dual Anxiety and Depression Diagnosis (Scores ≥ 10)**	285	18.85

**Table 3:** Descriptive statistics.

Variable				95% CI	
	Range	M	SD	LL	UL
Anxiety	0–21	6.43	4.94	6.19	6.68
Depression	0–27	7.79	5.48	7.51	8.06
Perceived Impact of Coronavirus on Life	5–25	15.86	4.17	15.65	16.07
Completing the Semester and Graduation	1–5	3.36	1.26	3.30	3.42
Finding A Job and Professional Development	1–5	3.67	1.20	3.61	3.73
Financial Situation	1–5	3.82	1.18	3.76	3.88
Relationships with Loved Ones, Family	1–5	2.44	1.43	2.37	2.51
Relations with Colleagues, Friends	1–5	2.57	1.36	2.50	2.64

**Table 4:** Means, standard deviations, and two-way ANOVA statistics for anxiety.

Variable	Female		Male		ANOVA			
	M	SD	M	SD	Effect	F (1, 1508)	*p*	η*_p_*^2^
PA Groups					S	41.27	<0.001	0.03
Inactive	6.58	4.98	4.87	4.52	PA	5.88	0.015	0.00
Active	7.29	4.96	5.49	4.67	S × PA	0.03	0.861	0.00

*n* = 1512. ANOVA = analysis of variance; S = sex, PA = physical activity.

**Table 5:** Means, standard deviations, and two-way ANOVA statistics for depression.

Variable	Female		Male		ANOVA			
	M	SD	M	SD	Effect	F (1, 1508)	*p*	η*_p_*^2^
PA groups					S	44.59	<0.001	0.03
Active	7.82	5.30	5.88	5.09	PA	10.33	0.001	0.01
Inactive	8.87	5.62	6.78	5.06	S × PA	0.06	0.812	0.00

*n* = 1512. ANOVA = analysis of variance; S = sex, PA = physical activity.

**Table 6:** Hierarchical regression results for anxiety.

Variable		95% CI for *B*					
	B	LL	UL	SE B	β	R^2^	ΔR^2^
**Step 1**						0.03 ***	0.03 ***
Constant	8.47 ***	8.15	8.80	0.17			
Sex	–2.17 ***	–2.78	–1.61	0.30	–0.19 ***		
**Step 2**						0.09 ***	0.06 ***
Constant	6.75 ***	6.26	7.24	0.25			
Sex	–2.11 ***	–2.68	–1.54	0.29	–0.18 ***		
Exposure to COVID–19	1.26 ***	0.99	1.53	0.14	0.23 ***		
**Step 3**						0.13 ***	0.04 ***
Constant	2.92 ***	1.88	3.95	0.53			
Sex	–2.21 ***	–2.77	–1.65	0.29	–0.19 ***		
Exposure to COVID–19	0.99 ***	0.72	1.27	0.14	0.18 ***		
Impact of COVID–19 (PCI)	0.27 ***	0.20	0.33	0.03	0.20 ***		
**Step 4**						0.14 ***	0.01 ***
Constant	4.42 ***	3.31	5.53	0.57			
Sex	–2.08 ***	–2.63	–1.53	0.28	–0.18 ***		
Exposure to COVID–19	0.99 ***	0.72	1.26	0.14	0.18 ***		
Impact of COVID–19 (PCI)	0.27 ***	0.21	0.34	0.03	0.21 ***		
Physical activity (PA)	–0.46 ***	–0.60	–0.33	0.07	–0.16 ***		

*** *p* < 0.001.

**Table 7:** Hierarchical regression results for depression.

Variable		95% CI for B					
	B	LL	UL	SE B	β	R^2^	ΔR^2^
**Step 1**						0.03 ***	0.03 ***
Constant	8.47 ***	8.14	8.80	0.17			
Gender	–2.20 ***	–2.78	–1.61	0.30	–0.19 ***		
**Step 2**						0.08 ***	0.05 ***
Constant	6.75 ***	6.27	7.24	0.25			
Gender	–2.11 ***	–2.68	–1.54	0.29	–0.18 ***		
Exposure to COVID–19	1.26 ***	0.99	1.53	0.14	0.23 ***		
Step 3						0.12 ***	0.04 ***
Constant	2.92 ***	1.88	3.95	0.53			
Gender	–2.21 ***	–2.77	–1.65	0.29	–0.19 ***		
Exposure to COVID–19	0.99 ***	0.72	1.27	0.14	0.18 ***		
Impact of COVID–19 (PCI)	0.27 ***	0.20	0.33	0.03	0.20 ***		
**Step 4**						0.15 ***	0.03 ***
Constant	4.42 ***	3.31	5.53	0.57			
Gender	–2.08 ***	–2.63	–1.53	0.28	–0.18 ***		
Exposure to COVID–19	0.99 ***	0.72	1.26	0.14	0.18 ***		
Impact of COVID–19 (PCI)	0.27 ***	0.21	0.34	0.03	0.21 ***		
Physical activity (PA)	–0.46 ***	–0.60	–0.33	0.07	–0.16 ***		

*** *p* < 0.001.
